# Supplementary figures and images for: Intestinal iron absorption is appropriately modulated to match physiological demand for iron in wild-type and iron-loaded Hamp (hepcidin) knockout rats during acute colitis
Source: PLoS One. 2021 Jun 18;16(6):e0252998. doi: 10.1371/journal.pone.0252998 (PMC8213193; doi:10.1371/journal.pone.0252998)

KO+DSS

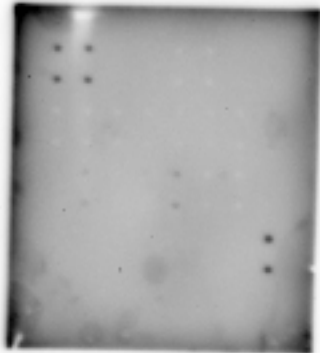

WT (no DSS)

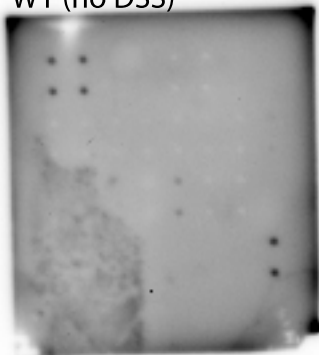

KO (no DSS)

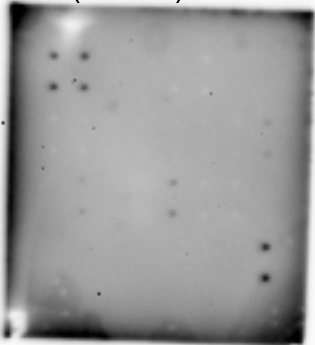

WT+DSS

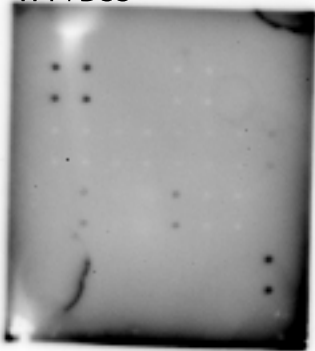

Supplement: S1 File — (PDF) [file pone.0252998.s001.pdf]

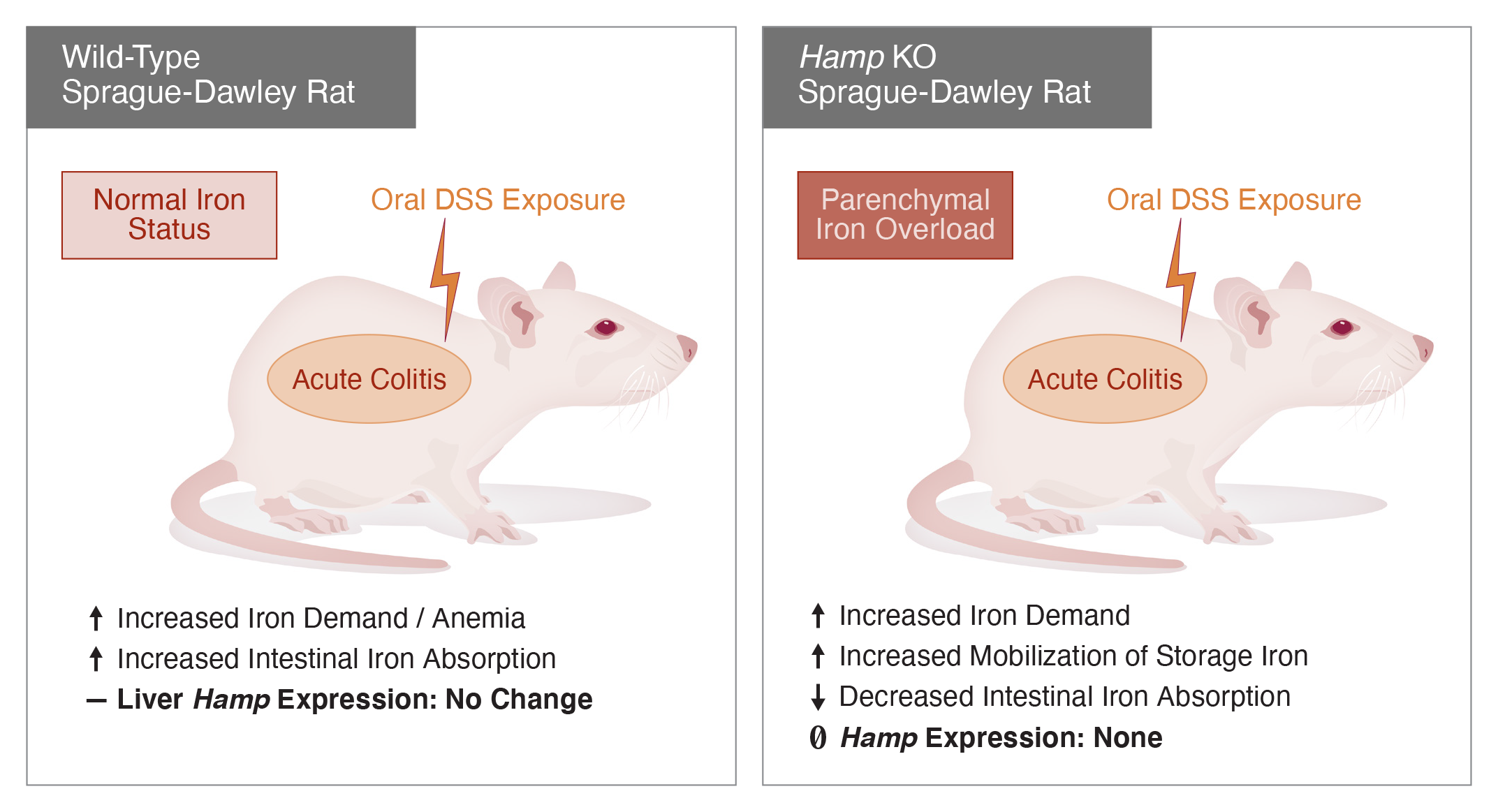

Supplement: S1 Graphical abstract — (TIF) [file pone.0252998.s002.tif]
